# Anatomical variations of pulmonary venous drainage in Thai people: multidetector CT study

**DOI:** 10.2349/biij.8.1.e4

**Published:** 2012-01-01

**Authors:** Y Wannasopha, N Oilmungmool, J Euathrongchit

**Affiliations:** Department of Radiology, Faculty of Medicine, Chiang Mai University, Chiang Mai, Thailand

**Keywords:** Multidetector CT, pulmonary vein, left atrium

## Abstract

**Objective:**

To evaluate the patterns of pulmonary venous drainage into the left atrium and to determine the frequency of each variant of pulmonary venous anatomy.

**Materials and methods:**

After institutional review board approval (No. 09JUL011148), 300 studies of thoracic multidetector computed tomography were retrospectively reviewed for the anatomical features of the pulmonary vein and its drainage pattern into the left atrium. The percentage of each pattern was calculated.

**Results:**

The anatomy of pulmonary venous drainage in 300 patients (150 male and 150 female, mean age 60.16 years) showed some variation. In the right pulmonary vein, the most common drainage pattern was two ostia (90.33%), followed by three to five ostia (6.33%) and a single ostium (3.33%). There were one or two separate middle lobe vein ostia in groups of more than two openings. On the left side, there were two patterns; a single venous ostium (59%) was much more common than two ostia (41%). In both right and left pulmonary veins, there were five cases (2 male, 3 female) that had a single pulmonary venous ostium, bilaterally. However, there were only 17 cases (5.67%), out of 300 enrolled in this study, that had bilateral pulmonary venous ostial variations.

**Conclusion:**

A classification system to succinctly describe pulmonary venous drainage patterns was developed. In left-sided drainage, a single left pulmonary ostium was the most common variation. The right-sided venous drainage varied more in both number and pattern than those of the left side; nevertheless, bilateral pulmonary venous ostial variation was not frequently found.

## INTRODUCTION

Generally, the body’s vasculature shows a different pattern in each individual, called normal variation. Likewise, the pulmonary vein also shows variations. This vein is an important source of ectopic atrial electrical activity, frequently initiating paroxysms of atrial fibrillation [1, 2]. Increasingly, selective radiofrequency ablation of these arrhythmogenic foci is performed to treat patients with refractory atrial fibrillation. The effectiveness of this invasive procedure relies on precise mapping and complete disconnection of the electrical initiators from the atrial tissue. Thus, detailed knowledge of pulmonary venous anatomy and relationships between the pulmonary veins and the left atrium is necessary during mapping and ablation [3]. Since there have been no previous studies looking at pulmonary venous drainage and its variation among Thai people, this study was carried out, using the modern and noninvasive method of multidetector computed tomography (MDCT), to find this data. The objectives of this study were to evaluate the patterns of pulmonary venous drainage into the left atrium and to determine the frequency of each variant of pulmonary venous amatomy among a Thai population.

## MATERIALS AND METHODS

### Study population

After institutional review board approval (No 09JUL011148), this retrospective study obtained data from the CT database for all patients who received thoracic MDCT scans at the authors’ institution, with various indications such as lung cancer, chronic cough, chest pain, dyspnoea, etc, from February 1, 2008 to June 30, 2009.

There were 2,290 cases (N) that underwent thoracic MDCT scans in that period. Cases in which CT images showed poor pulmonary vein enhancement, distortion of the pulmonary vein by mediastinal or lung mass, or destroyed lung were excluded. The sample size (n) was calculated using the Taro Yamane equation (n = N/ [1+N (e) 2]; N = 2,290, e = 0.0540). Finally 300 cases were enrolled and studied for age, sex, and pulmonary venous drainage pattern.

### CT data set

All CT examinations were obtained by dual source MDCT scanner (Somatom Definition; Siemens, Forchheim, Germany) or 16-channel scanner (Aquilion 16, Toshiba, Tochigi-Ken, Japan) with patient in the supine position and holding a deep breath. The CT data set for each case was taken by one of the three major acquisition protocols: 1) Contrast-enhanced conventional CT chest protocol, 2) CT angiography (CTA) to evaluate the pulmonary thromboembolism (CTA – PE) protocol, and 3) CT angiography of the coronary artery.

As there were many series in each protocol, only data sets that covered the lung apices to the bases, with 1 mm section thickness and interval in conventional CT chest, and CTA – PE protocols and data sets that covered the aortic arch to the diaphragm with 0.75 mm slice thickness and spacing in CTA coronary artery protocol were selected. Each CT data set was reviewed by a third-year resident (NO) and two radiologists (YW and JE), who have worked in thoracic imaging for 2 years and 10 years, respectively.

Soft copy DICOM images were retrieved from a picture archiving and communication system (PACS). Axial and multiplanar reformatted (MPR) images were reviewed with workstations (Panacea, version 2.0.1, Bangkok, Thailand and Merge E-film, version 1.9.4, Toronto, Ontario, Canada). Three-dimensional (3D) volume rendering images were done using an advanced workstation (Voxar 3D, version 4.1SR2; Voxar, Framingham, Massachusetts, US) and evaluated for 3D pulmonary vein anatomy and drainage orifice in each type.

### Pulmonary vein classification and statistical analysis

**Figure 1:**
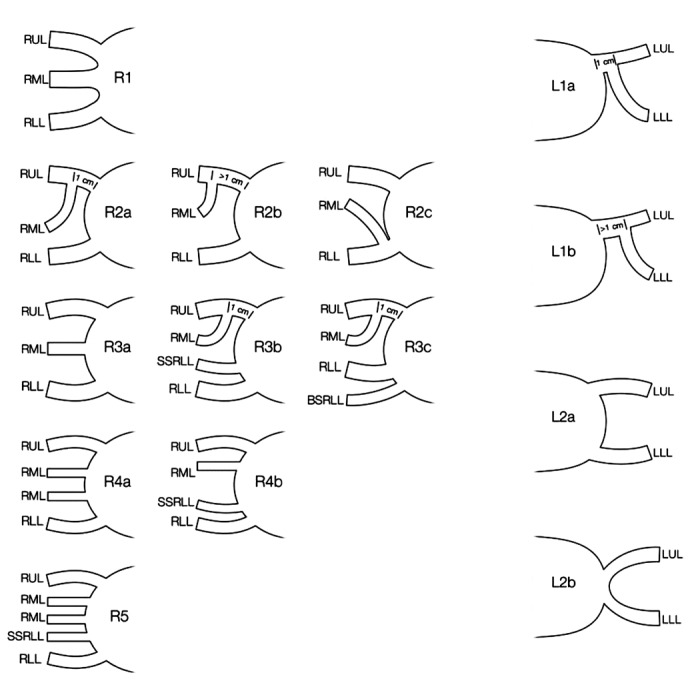
Modified diagram of pulmonary venous origin category by Marom *et al*. [1]. *BSRLL = *basilar segment right lower lobe pulmonary vein, *RLL = *right lower lobe pulmonary vein, *RML = *right middle lobe pulmonary vein, *RUL = *right upper lobe pulmonary vein, *SSRLL = *superior segment right lower lobe pulmonary vein, *LUL* = left upper lobe pulmonary vein, *LLL* = left lower lobe pulmonary vein, *1cm = *distance from the ostium.

**Figure 2:**
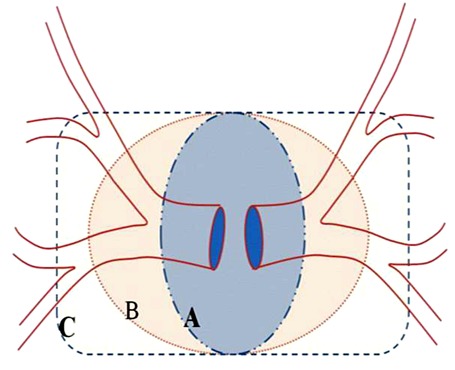
Modified diagram of three variant configurations of the atrial-venous junction by Ghaye B *et al*. [3]. The pulmonary veins are the red lines, and the oval (A & B) and rectangular (C) shapes represent the left atrium. A normal pulmonary venous ostial pattern is shown in image B, which has two ostia on each side. Under-incorporation or over-incorporation of the pulmonary veins into the left atrium are demonstrated in A and C, respectively. Different cardiac development results in variable lengths of the common trunk, and variable numbers and morphology of the ostia and the pulmonary venous branches.

Marom EM *et al.* divided the pulmonary vein and its drainage orifices into 6 patterns on the right side and 2 patterns on the left side [1] (Figures 1 and 2). This system was used to classify the pulmonary venous drainage pattern and images differing from this arrangement were also identified. All data (age, gender, CT technique, and pulmonary venous pattern) were recorded and calculated for the percentage of each pulmonary venous drainage type using a computer spreadsheet (Excel 2003; Microsoft, Redmond, Washington, US).

## RESULTS

A total of 150 male and 150 female patients were studied. Ages ranged from 14 to 94 years with a mean age of 60.16 years. Data sets were obtained with conventional CT chest technique in 90 studies, CTA – PE in 117 studies, and CTA coronary artery in 93 studies. Using Marom’s pulmonary venous drainage categories, the drainage patterns were summarised in Table 1 for the right pulmonary vein and Table 2 for the left pulmonary vein.

**Table 1:** Right pulmonary venous drainage patterns.

**Drainage pattern**	**Description**	**No. of patients**	**Percent**
R1	Single common ostium in the left atrium receiving upper, middle, and lower lobe veins	10	3.33
R2a	Two atrial ostia for the upper and lower lobe veins; the middle lobe vein joins the proximal upper lobe vein less than 1 cm from the ostium	47	15.67
R2b	Two atrial ostia for the upper and lower lobe veins; the middle lobe vein joins the proximal upper lobe vein more than 1 cm from the ostium	217	72.33
R2c	Two atrial ostia for the upper and lower lobe veins; the middle lobe vein joins the lower lobe vein	6	2
R3a	Three atrial ostia for the upper, middle, and lower veins	6	2
R3b	Three atrial ostia for the upper, superior segment, and lower lobe veins; the middle lobe vein joins the proximal upper lobe vein less than 1 cm from the ostium	6	2
R3c	Three atrial ostia for the upper, a basilar right lower lobe vein, and lower lobe veins; the middle lobe vein joins the proximal upper lobe vein less than 1 cm from the ostium	0	0
R4a	Four atrial ostia for one upper, two middle, and one lower lobe veins	3	1
R4b	Four atrial ostia for the upper, middle, superior segment, and lower lobe veins	1	0.3
R5	Five atrial ostia for one upper, two middle, two superior segment, and one lower lobe veins	0	0
Others	Different in lobar vein opening and ostia	4	1.3
Total		300	100

**Table 2:** Left pulmonary venous drainage patterns.

**Drainage pattern**	**Description**	**No. of patients**	**Percent**
L1a	Lower lobe vein joins upper lobe vein to form a common trunk vein less than 1 cm long that drains into the left atrium (one ostium)	74	24.67
L1b	Lower lobe vein joins upper lobe vein to form a common trunk vein more than 1 cm long that drains into the left atrium (one ostium)	103	34.33
L2a	Two atrial ostia for the upper and lower lobe veins, respectively; ostia are separated by left atrial wall	76	25.33
L2b	Two atrial ostia for the upper and lower lobe veins, respectively; ostia are not separated by left atrial wall	47	15.67
Total		300	100

**Figure 3:**
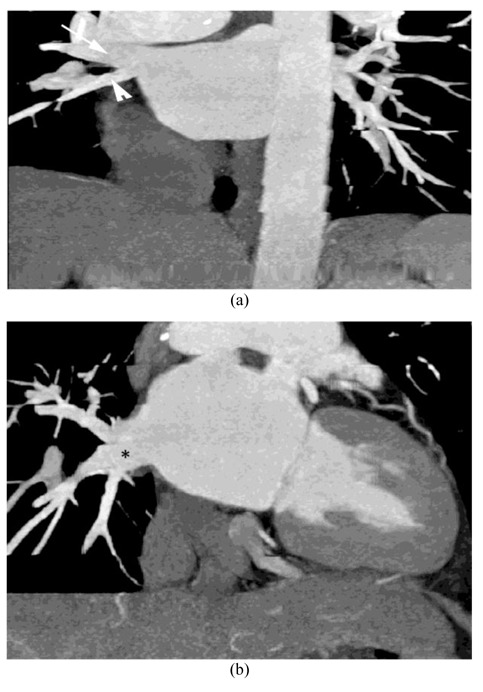
R2a pattern: Thick-slab coronal oblique (A) and coronal MPR images (B) of a 58-year-old woman showed atrial ostia for the upper (arrow) and lower lobe (arrowhead) veins; the middle lobe vein (asterisk) joins the proximal upper lobe vein less than 1 cm from the ostium.

**Figure 4:**
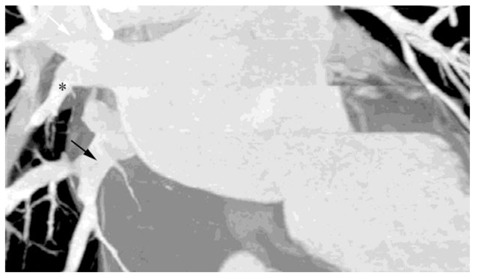
R2b pattern: Thick-slab coronal oblique MPR image of a 65-year-old woman showed two atrial ostia for the upper (white arrow) and lower lobe (black arrow) veins; the middle lobe vein (asterisk) joins the upper lobe vein more than 1 cm from the ostium.

Right pulmonary venous drainage patterns (Table 1): Most patients (n = 264, 88%) had the expected anatomy of two atrial ostia for upper and lower lobe veins, with the middle lobe vein joining the upper lobe vein (Figures 3 and 4). The remaining patients (n = 36, 12%; male = 24, female = 12) had other variant anatomy.

**Figure 5:**
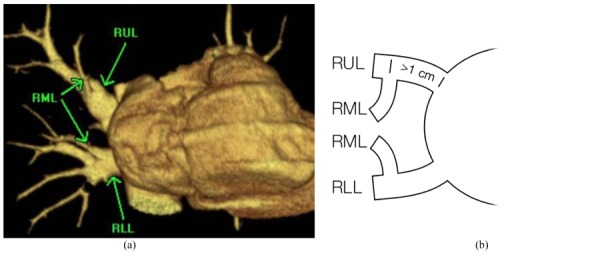
Other form: 3DCVR (A) and diagram (B) of a 47-year-old man demonstrated two atrial ostia for the upper and lower lobe veins; one middle lobe vein joins the proximal upper lobe vein more than 1 cm from the ostium and the other one joins the lower lobe vein.

**Figure 6:**
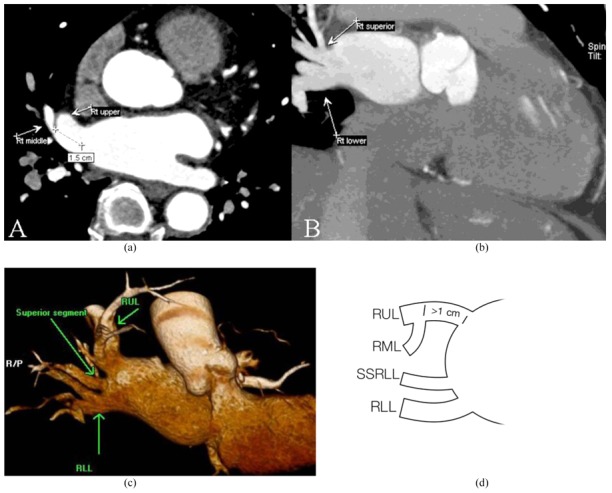
Other form: Axial image (A), thick-slab coronal oblique MPR image (B), 3DVR image (C) and diagram (D) of a 71-year-old woman revealed three atrial ostia for the upper, superior segment, and lower lobe veins; the middle lobe vein joins the proximal upper lobe vein more than 1 cm from the ostium.

**Figure 7:**
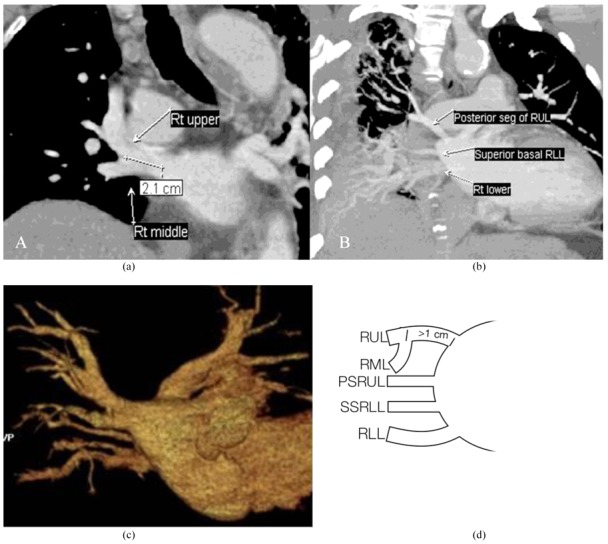
Other form: coronal image (A), thick-slab coronal oblique MPR image (B) of a 57-year-old man showed four atrial ostia for the upper, posterior segment upper lobe, superior segment lower lobe, and lower lobe veins; the middle lobe vein joins the proximal upper lobe vein more than 1 cm from ostium. 3DVR images and diagram (C and D) also demonstrated the four ostia.

Four cases that differed from Marom’s classification were found. The first patient showed two ostia for the upper and lower lobe veins opening to the left atrium with two middle lobe veins. One branch joined the proximal upper lobe vein more than 1 cm from the ostium and the other branch joined the lower lobe vein (Figure 5). In the next two cases, their CT demonstrated three ostia for upper, superior segment of lower lobe, and lower lobe veins with the middle lobe vein, joining the proximal upper lobe vein more than 1 cm from the ostium (Figure 6). The last case had four ostia for the upper, posterior segment of the upper lobe, superior segment of the lower lobe, and lower lobe veins; the middle lobe vein joined the proximal upper lobe vein more than 1 cm from the ostium (Figure 7).

**Figure 8:**
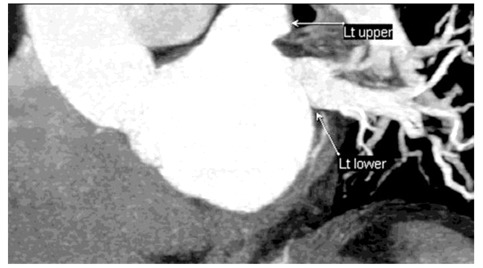
: L2a pattern: Thick-slab coronal oblique MPR image of a 61-year-old man showed two atrial ostia for the upper and lower lobe veins; ostia are separated by the left atrial wall.

**Figure 9:**
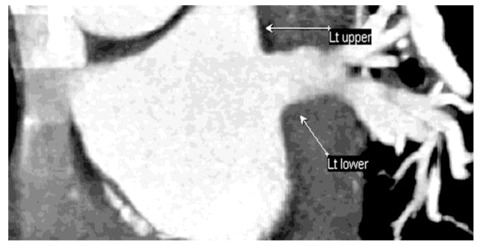
: L2b pattern: Thick-slab MPR image of a 54-year-old man showed two atrial ostia for the upper and lower lobe veins; ostia are not separated by the left atrial wall.

**Figure 10:**
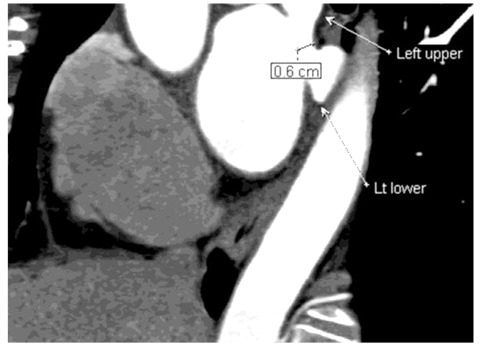
L1a pattern: Left anterior oblique coronal image of a 60-year-old man revealed the lower lobe vein joining the upper lobe vein to form a common trunk vein less than 1 cm long that drains into the left atrium (one ostium).

**Figure 11:**
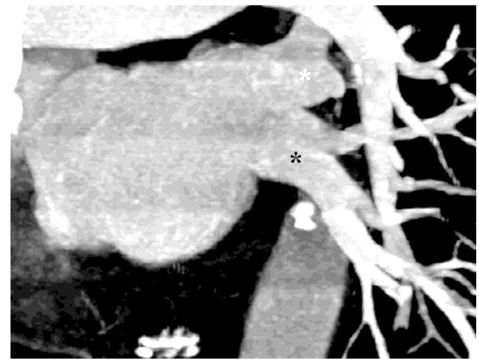
L1b pattern: Thick-slab coronal oblique MPR image of a 69-year-old woman showed lower lobe vein (black asterisk) joining the upper lobe vein (white asterisk) to form a common trunk vein more than 1 cm long that drains into the left atrium (one ostium).

Left pulmonary venous drainage patterns (Table 2): No cases differed from Marom’s classification of the left pulmonary venous ostium. There were 123 (41%) patients with two ostia for the upper and lower lobe veins (Figures 8 and 9). A common trunk forming one ostium in the left atrium was seen in 177 (59%) patients (Figures 10 and 11), which was a slightly higher percentage than the patients with two ostia. Female patients (91 cases) had a single ostium much more often than male patients (86 cases).

Both right and left pulmonary veins: Five cases (2 male, 3 female) showed a single pulmonary venous ostium, bilaterally. There were 17 of 177 cases of single left pulmonary venous ostium that had right pulmonary venous ostial variation. Of 36 cases with right pulmonary variation, only 5 patients had a single left ostium. Total variations of pulmonary ostia were 196 (65.33%) out of 300 cases enrolled in this study [17 cases (8.67%) showed variation of ostia on both sides, 19 cases (9.69%) had variation only on the right, and 160 cases (81.63%) demonstrated variation only on the left]. Thus, there were only 17 cases (5.67%) from 300 cases enrolled in this study that showed bilateral pulmonary venous ostial variations.

## DISCUSSION

Atrial fibrillation is the most common cause of cardiac arrhythmia and produces significant morbidity and mortality [1, 3]. The ectopic beat commonly originates in the ostia of the pulmonary vein. Electrocardiography is the classic diagnostic tool for this condition. There are many methods for treatment, starting with anti-arrhythmic medications, cardioversion or pacemaker, surgical procedure, and percutaneous ablation [2, 3]. Since ablation therapy is increasingly performed, radiologists should be familiar with the normal and anatomical variations of the pulmonary vein.

Pulmonary venous variations or abnormalities are related to the complex development of the venous system. In the embryo, pulmonary venous drainage is via the splanchnic plexus into the primordium of the systemic venous system, through both the cardinal and the umbilicovitelline veins [4]. The cardiac sinoatrial regions have caudal and cranial outpouchings that grow toward the lung buds. While the caudal portion regresses, the cranial portion continues to form the common pulmonary vein. By 28–30 days of gestation, the cranial portion communicates with the pulmonary venous plexus, which is now being separated from the main splanchnic plexus [4]. By 30–32 days, most connections with the splanchnic plexus are lost and the common pulmonary vein becomes incorporated into the left atrial wall, with the four pulmonary veins separately entering the chamber [4].

Approximately 70% of the general population have four pulmonary veins: right superior and inferior pulmonary veins and left superior and inferior pulmonary veins, with four independent ostia [1, 3, 5]. The superior pulmonary veins enter the mediastinum downward and anterior to their accompanying pulmonary arteries. The left superior vein is longer than the right vein. The inferior pulmonary veins are oriented upward and located below their homonymous bronchi. Again, anatomical variations in the number, branching patterns, and length of preostial portions of the pulmonary veins, which occur during gestation, result from the under-incorporation or over-incorporation of the common pulmonary vein into the left dorsal atrium as shown in Figure 2 [3].

In this study, most patients (90.3%) had two ostia on the right side for upper and lower lobe veins, while 41% of patients had normal two-ostial patterns on the left. In the literature that was reviewed, variation of right pulmonary venous drainage and ostia were much more common than the left [1, 5]. However, in this study, it was found that most cases showed a single pulmonary ostium on the left (59%), even as the right side had more variation patterns than the left.

There were 10 patients (3.33%) with a single venous ostium on the right side and 177 patients (59%) who had one venous ostium on the left side. Under-incorporation may be asymmetric and involve the confluence of either superior pulmonary veins or both inferior pulmonary veins, or of the superior and inferior pulmonary veins on one side (Figure 2). The latter appeared as a single ostium and is present in 12–25% of the general population, usually on the left side [3], as in this study (single left pulmonary ostium – 59%).

Supernumerary or accessory pulmonary veins with their own ostia occur by over-incorporation beyond the first division of the pulmonary vein (Figure 2). An increase in the number of pulmonary veins usually shows on the right side [3]. This study also found 14 cases in which the opening had three ostia and 5 cases in which the opening had four ostia on the right, which occurred infrequently [3]. There was no case of supernumerary pulmonary vein on the left side found in this study.

Total variation of this study was higher than in the literature, and bilateral pulmonary venous ostial variation was about 5.67%. There were four cases that could not be classified using the categories of Marom EM [1] and were found only on the right side. One patient had two ostia for the upper and lower lobe veins opening to the left atrium with two middle lobe veins; each middle lobe vein joined the upper and lower lobe veins. The next two patients had three ostia for upper, superior segment and lower lobe veins; the middle lobe vein joined the proximal upper lobe vein more than 1 cm from the ostium. The other patient had four ostia for the upper, posterior segment upper lobe, superior segment, and lower lobe veins; the middle lobe vein joined the proximal upper lobe vein more than 1 cm from the ostium.

## CONCLUSION

This study confirmed that the most frequent pattern of pulmonary venous drainage on the right side was two ostia into the left atrium (90.3%). However, a higher incidence of pulmonary variation was found in the Thai population than what was reported in the literature. The most common pulmonary venous variation was single left pulmonary ostium (59%). The right-sided venous drainage varied more in both number and pattern than those of the left side; nevertheless, bilateral pulmonary venous ostial variation was not frequently found, at about 5.67%.
